# Effect of Otago Exercise Program Combined with Neuromuscular Electrical Stimulation on Chronic Ankle Instability in Older Adults: A Pilot Randomized Controlled Trial

**DOI:** 10.3390/jcm15051968

**Published:** 2026-03-04

**Authors:** Yunong Zhang, Min-Chul Shin, Ye Tao, Kexiang Yang, Shuting Liu

**Affiliations:** 1 Department of Physical Education, Sejong University, Seoul 05006, Republic of Korea; 2 Department of Physical Education, Jeonbuk National University, Jeonju 54896, Republic of Korea

**Keywords:** chronic ankle instability, Neuromuscular Electrical Stimulation, Otago Exercise Program, older adults with CAI

## Abstract

**Background**: Chronic ankle instability (CAI) is a common functional disorder in older adults, affecting their balance and quality of life. Therefore, finding effective ways to enhance ankle stability and function under safe conditions remains a key issue for healthy aging. **Objective**: This study aims to explore the effects of the Otago Exercise Program (OEP) combined with Neuromuscular Electrical Stimulation (NMES) on ankle stability, the pain index, and balance ability in older adults aged 60 and above with CAI. **Methods**: This study is a single-blind pilot randomized controlled trial, including 36 eligible older adults with CAI, with 34 completing the trial. Participants were randomly assigned to the OEP group, the combined group (OEP + NMES), and the control group. The intervention period lasted 8 weeks. Evaluation measures included the Cumberland Ankle Instability Tool (CAIT), Visual Analog Scale (VAS), Eyes-closed Single-Leg Stance Test (UST), and the Modified Star Excursion Balance Test (mSEBT), with assessments conducted before the intervention, at week 4, and at week 8. **Result**: After the intervention, all three groups showed significant improvements in CAIT, VAS, UST, and mSEBT scores (*p* < 0.05), with a large group × time effect observed for the primary outcome CAIT (partial η^2^ = 0.414). The combined group (OEP + NMES) demonstrated the most significant improvement in CAIT and UST scores (*p* < 0.05) and outperformed the other two groups in dynamic balance in the posteromedial and posterolateral directions. **Conclusions**: The combined intervention of OEP and NMES significantly improves ankle stability, both static and dynamic balance abilities, and alleviates pain in older adults with CAI. This combined approach offers a safe and effective rehabilitation strategy for the older adults, with promising clinical application prospects.

## 1. Introduction

With the aging population, health issues among the older adults have increasingly become a significant concern in global public health, particularly chronic ankle instability (CAI), which has become a common functional disorder in older adults. It has been reported that the incidence of chronic ankle instability among individuals aged 60 and above worldwide has reached 7% [1]. Older adults are prone to CAI due to age-related declines in proprioception, muscle strength, joint stability, balance control, and delayed neuromuscular responses [2]. For older adults, ankle instability increases the risk of falls and other injuries and is also associated with proprioceptive deficits, impaired static and dynamic balance, reduced ankle range of motion, and decreased muscle strength, even severely impacting their daily lives [3].

Traditional rehabilitation for CAI commonly emphasizes balance- and strength-oriented exercise. Evidence shows that balance training can enhance sensorimotor control and functional performance and may also benefit muscle strength and mobility-related outcomes in individuals with CAI [4].

Currently, there are several treatment options for this condition. Traditional treatments mainly focus on balance training and strength training [5].

The Otago Exercise Program (OEP), as a form of exercise therapy, has gained increasing attention in recent years. The OEP is a lightweight exercise program specifically designed for older adults, aimed at improving balance, gait, and strength to enhance physical function in this population [6]. Research has shown that the OEP can increase muscle strength and improve static and dynamic balance control, thereby significantly enhancing ankle stability [7]. However, other researchers have disputed this conclusion. In a randomized controlled trial, Willems found that the OEP had no significant impact on improving static balance, as it alone could not address the issues of static balance and ankle proprioception deficits [8]. Sahin argued that the OEP alone is insufficient to stimulate ankle proprioceptors [9]. These results indicate that a single rehabilitation program may not provide significant short-term benefits, especially for older adults, whose physiological characteristics and limitations in physical ability restrict treatment effectiveness. Therefore, researchers need to consider more effective ankle-rehabilitation exercises and plans, such as combined treatments involving multiple interventions.

Neuromuscular Electrical Stimulation (NMES), a therapeutic method that stimulates muscles through electrical currents to promote muscle contraction, has gained widespread use in rehabilitation medicine in recent years [10]. NMES stimulates deep muscle groups with electrical currents, enhancing muscle endurance [11], helping older adults regain muscle strength, especially in the muscle groups around the ankle joint [12]. Studies have shown that NMES can improve the activity of deep muscles, enhance muscle function, and reduce the joint instability caused by muscle weakness [13].

This study aims to evaluate the efficacy of the combined Otago Exercise Program (OEP) and Neuromuscular Electrical Stimulation (NMES) in the treatment of chronic ankle instability (CAI), pain, and balance in older adults. Through this research, we hope to provide a more efficient and comprehensive rehabilitation treatment plan for older adults with CAI, while filling the gap in the existing literature regarding the combined use of these two methods. This study will further verify the efficacy of OEP combined with NMES by comparing the effects of different interventions, providing new practical guidance for the rehabilitation of older adults with CAI.

The following hypothesis is proposed in this study. The combined intervention of the Otago Exercise Program and Neuromuscular Electrical Stimulation will be more effective than either Otago Exercise Program alone or conventional rehabilitation training in improving ankle stability, the pain index, and balance ability in older patients with chronic ankle instability (CAI).

## 2. Materials and Methods

### 2.1. Participants

This study established the inclusion and exclusion criteria based on the standards set by the International Ankle Instability Association (Gribble, 2013) [14]. To further investigate chronic ankle instability and its treatment, older adults aged 60 and above from the Taikang Nursing Home in Jinan were selected as the study subjects. Older adults with chronic ankle instability or recurrent sprains were included. Prior to the experiment, participants were required to undergo the single-leg stance [15], Trendelenburg test [16], dynamic balance test [17], anterior drawer test for ankle forward displacement [18], and Y-balance test [19]. If participants had positive results in two or more of these tests [20], they were diagnosed with chronic ankle instability and met the criteria for inclusion in the experiment.

### 2.2. Randomization and Concealment

This study employed an assessor-blinded, single-blind pilot randomized controlled design. Group allocation was performed by researchers independent of the outcome assessment and data analysis. Outcome evaluators were blinded to the group assignment throughout the study. Due to the nature of the exercise interventions, participants and intervention providers could not be fully blinded. Data were collected using coded identifiers, and group allocation remained concealed until completion of the final analysis [21]. The random allocation sequence was generated using a computer-based random-number generator (1:1:1 ratio). Assignments were placed in sequentially numbered, sealed, opaque envelopes prepared by an independent researcher not involved in recruitment, assessment, or analysis.

These methodological safeguards were applied to improve internal validity and reduce potential sources of bias. A total of 36 eligible participants were recruited for the experiment, and all signed informed consent forms. The study was approved by the Ethics Committee of Sejong University (Approval Number: SUIRB-HR-2015-015), in accordance with the Declaration of Helsinki. The trial was registered with cris.nih.go.kr prior to study recruitment (ID: KCT0010816).

### 2.3. Inclusion Criteria

(1) Age Range: Participants are older adults aged 60–75 years.
(2) Injury and Health Condition: Participants must have experienced chronic ankle instability symptoms for more than 3 months, with stable symptoms that meet the definition of a chronic condition and have a CAIT score of 24 or below. Overall health should be good, with no severe ankle joint injuries or other serious diseases that could affect the execution of the experiment (such as severe respiratory, cardiovascular, or neurological diseases).
(3) Physical Ability: Participants must have sufficient physical capacity to complete exercises of moderate intensity and duration and should be able to walk at least 20 m without the need for assistive devices (such as canes, walkers, etc.).
(4) Voluntary Participation: All participants must voluntarily participate and sign an informed consent form.
(5) Cognitive Ability: Participants must have sufficient cognitive function to understand the experimental requirements and training content.

### 2.4. Exclusion Criteria

(1) Surgical History: Participants with a history of ankle surgery were excluded because surgical intervention can alter ankle structure and function.
(2) Severe Trauma History: Participants with recent severe ankle injuries, such as sprains or fractures, that may affect the rehabilitation process and outcome evaluation.
(3) Severe Comorbidities: Conditions such as heart disease, uncontrolled hypertension, or diabetes, which may limit the participant’s physical abilities or increase risks during the experimental process.
(4) Compliance: Participants who are expected to be unable to consistently follow the study or complete the experiment.
(5) Cognitive Ability: Participants with cognitive impairments who are unable to understand or follow the study procedures.

### 2.5. Procedures

A total of 36 older adults voluntarily participated in this study. Based on the results of the CAIT (Table 1), participants were randomly assigned into three groups: Otago Exercise Program (OEP) group (*n* = 12), OEP + Neuromuscular Electrical Stimulation (NMES) combined group (*n* = 12), and control group (*n* = 12).

**Figure 1:**
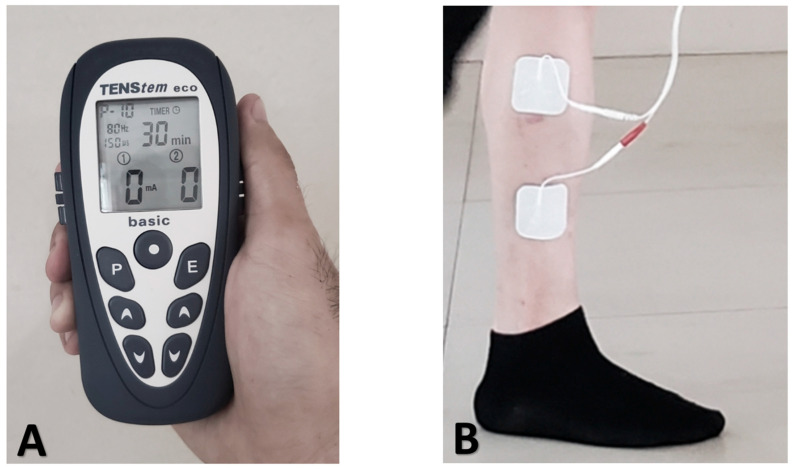
(**A**) Neuromuscular Electrical Stimulation device; Product Name: TENStem eco BASIC (JIAJIAN Corp., Wuxi, Jiangsu, China); (**B**) electrodes attached to the skin.

The OEP group performed the OEP three times a week. In contrast, the OEP + NMES group combined the OEP with NMES twice a week (Figure 1). The control group received conventional rehabilitation training. The study was completed over 8 weeks, with measurements taken before the intervention, at week 4, and at week 8. The training volume was calculated by multiplying the total number of OEP sessions completed by both the OEP and OEP + NMES groups during the 8-week training period by the number of repetitions per session. When calculating the training volume, the researchers observed the steps described in the exercise program section and recorded the repetitions completed successfully by the participants. To determine the specific effect of the OEP in this study, the training load and rest intervals for all participants in the conventional training group were set to be similar to each other (Table 1).

Participants in the control group received a standardized conventional rehabilitation program focusing on ankle mobility, low-load strengthening, and basic balance exercises, without structured Otago exercises or NMES. Training was conducted three times per week for 8 weeks, matching the overall training duration of the intervention groups. The OEP group performed three sessions/week (≈30–35 min/session; ≈90–105 min/week). The OEP + NMES group completed the same OEP dose plus NMES delivered in separate sessions twice/week (20 min/session; 40 min/week). The control group trained three sessions/week (≈30–35 min/session; ≈90–105 min/week). In addition, the OEP and NMES was delivered separately.

Each session included the following: (1) warm-up (5 min), light walking and active ankle range-of-motion exercises (dorsiflexion, plantarflexion, inversion, eversion); (2) strengthening (15–20 min), seated or standing ankle plantarflexion and dorsiflexion exercises, resistance-band exercises for ankle invertors and evertors (2–3 sets of 10–12 repetitions). Exercise intensity was maintained at a low-to-moderate level, adjusted to participant tolerance to ensure safety.

**Table 1:** Combined training program.

Week	Otago Exercise Content	NMES	Notes
1	Daily Warm-up: 5 min of light walking Balance Exercise: Single-leg stance (Figure 2) (2 times per leg, 30 s each) Gait Training: Heel-to-toe walking	Twice a week, 20 min each time, moderate intensity, focusing on the muscle groups around the ankle joint	Adaptation phase, gradually increase exercise volume and NMES time
2	Warm-up as above; Balance Exercise: Single-leg stance with eyes closed (2 times per leg, 20 s each) Strengthening Exercise: Seated hip raise (2 sets, 10 reps each)	Same as above, but intensity can be increased moderately to ensure comfort	Adjust exercise difficulty based on individual conditions
3–4	Warm-up as above; Balance Exercise: Increase use of balance board for practice (30 s each time, 2 sets) Dynamic Exercise: Side stepping (10 steps per side)	Continue NMES treatment daily, intensity adjusted based on body response	Add more challenging balance exercises
5–6	Warm-up as above; Balance Exercise: Further challenge by standing on a foam pad, single-leg stance (30 s each time, 2 sets per leg) Dynamic Strengthening: Standing hip raise (2 sets, 10 reps each)	Maintain NMES, encourage participants to engage in light activity during non-NMES periods	Increase balance and strength requirements
7–8	Warm-up as above; Balance Exercise: Combine dynamic movements such as forward and backward straight-line walking (10 steps in each direction) Strengthening Exercise: Add side stepping with resistance band assistance (10 steps per side)	Maintain NMES, encourage participants to engage in light activity during non-NMES periods	Prepare for the final evaluation, emphasizing integrated abilities

The setting of NMES was as follows: biphasic symmetrical waveform; frequency, 35 Hz; pulse duration, 300 μs; duty cycle, 5 s on/10 s off; session duration, 20 min; intensity adjusted to visible, comfortable muscle contraction.

### 2.6. Outcome Measures

The primary outcome measure was the Cumberland Ankle Instability Tool (CAIT). Secondary outcome measures included the Visual Analog Scale (VAS), Eyes-closed Single-Leg Stance Test (UST), and modified Star Excursion Balance Test (mSEBT).

#### 2.6.1. Subjective Instability Sensation/Cumberland Ankle Instability Tool (CAIT)

Subjective instability sensation was assessed using the Cumberland Ankle Instability Tool (CAIT), an effective and reliable assessment tool (ICC = 0.96) [22]. The questionnaire comprises nine items, yielding a total score from 0 to 30, with lower values reflecting poorer ankle stability.

#### 2.6.2. Visual Analog Scale (VAS)

The Visual Analog Scale (VAS) is used to quantify the degree of pain perceived by the patient and is particularly suitable for assessing pain in postoperative patients [23]. Pain, being a subjective experience, varies in sensitivity among individuals, which can influence a physician’s judgment of the patient’s condition. In this study, the VAS was used to assess ankle joint pain, with the aim of quantifying the patient’s subjective pain experience to indirectly reflect the progression of the underlying disease. A score of 0 indicates no pain, while a score of 10 indicates severe pain. Detailed records of pain scores at different stages following ankle surgery help observe rehabilitation outcomes and provide guidance for rehabilitation exercises.

#### 2.6.3. Eyes-Closed Single-Leg Stance Test (UST)

**Figure 3:**
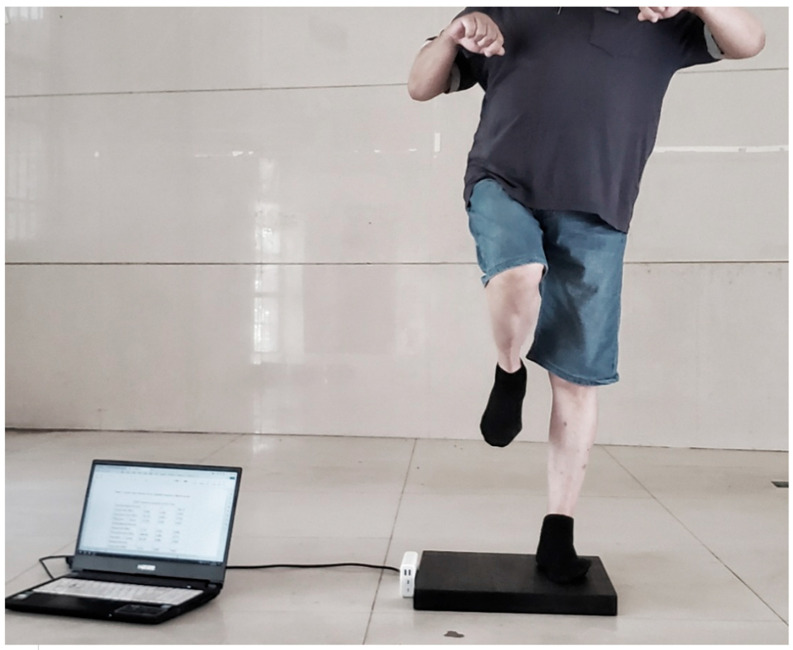
Eyes-closed Single-Leg Stance Test.

The Eyes-closed Single-Leg Stance Test (UST) is used to measure and estimate the static balance of participants [24]. In this test, participants were instructed to stand barefoot, eyes closed, with arms crossed over the chest and feet aligned forward (Figure 3) (for illustration purposes only. During actual testing, participants were instructed to keep their arms crossed over the chest, as described in the Methods section). When the start command is given, the participants close their eyes and use the affected leg as the supporting leg while raising the opposite leg so that the foot is at knee height of the supporting leg, holding the position until they lose balance. The effective time for the single-leg stance is measured with a stopwatch, starting from the start command. The test is stopped under any of the following conditions: (1) arms are not crossed over the chest; (2) the non-supporting leg touches the ground or moves away from the supporting leg; (3) the supporting leg shifts position; (4) eyes are opened; (5) the stance time reaches 45 s. Each participant performs 3 repetitions, with a 20 s rest between each trial. The average of the three tests is used as the participant’s final score for statistical analysis. The UST has been shown to have high test–retest reliability (ICC = 0.998).

#### 2.6.4. Star Excursion Balance Test (SEBT)

**Figure 4:**
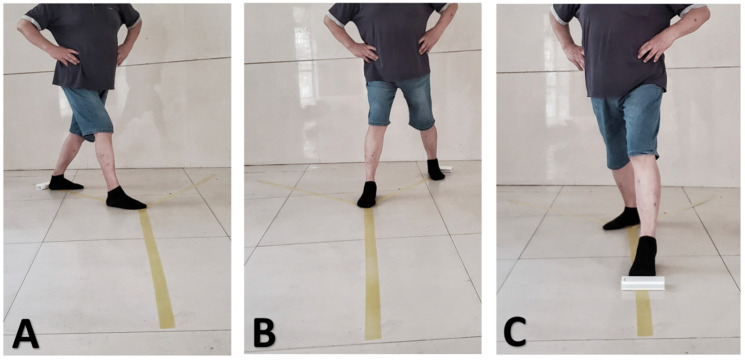
The modified Star-Excursion Balance Test. (**A**) Posteromedial direction; (**B**) posterolateral direction; (**C**) anterior direction.

The Star Excursion Balance Test (SEBT) is used to measure and estimate dynamic balance in participants [25]. The SEBT includes a combination of strength, flexibility, and coordination. In this study, a simplified version of the test with three directions was used based on previous research. During the test, participants are required to stand on one leg, maintaining balance while using the affected leg for support. The non-supporting leg is then extended as far as possible in three directions: posterior–medial, posterior–lateral, and forward (Figure 4). After each extension, the participant returns the non-supporting leg to the starting position. A trial was considered invalid if the stance foot shifted or the reaching limb contacted the ground. The maximal reach distance (cm) in each direction was recorded as the outcome measure. Participants completed four practice trials before testing, followed by three recorded trials per direction, separated by 20 s rest intervals. The mean of the three trials was used for analysis. The relative distance in each direction is used as the evaluation metric, and the participant’s total score is calculated as [(sum of the maximum values in the three directions)/(3 times leg length)] × 100. The SEBT has been proven to be an effective method for evaluating ankle stability, with good internal reliability (ICC = 0.86–0.94) and test–retest reliability (ICC = 0.89–0.93).

### 2.7. Statistical Analysis

Data from this study were analyzed and processed using SPSS 26.0 statistical software (IBM Corp., Armonk, NY, USA). One-way multivariate analysis of variance (ANOVA) was used to evaluate participants’ baseline characteristics. The four dependent variables were the subjective instability sensation (CAIT), Visual Analog Scale (VAS), Eyes-closed Single-Leg Stance Test (UST), and Star Excursion Balance Test (SEBT). For quantitative data meeting normal distribution criteria, a two-way ANOVA with repeated measures was performed at three time points, analyzing all data. The model included one between-subjects factor (treatment group with 3 levels: OEP group, combined group, and control group) and one within-subjects factor (time: baseline, week 4, and week 8). Bonferroni post-hoc tests and pairwise multiple comparisons were used to determine which changes between treatment groups (differences between pre- and post-training) were significant. Given the exploratory nature of secondary outcomes, no additional adjustment across outcome domains was applied. These change values were analyzed using one-way ANOVA, with the change values as the dependent variable and the three treatment groups as the grouping factor in the analysis. An alpha level of *p* < 0.05 was determined to be significant for all statistical comparisons.

## 3. Results

### 3.1. Baseline Characteristics

**Figure 5:**
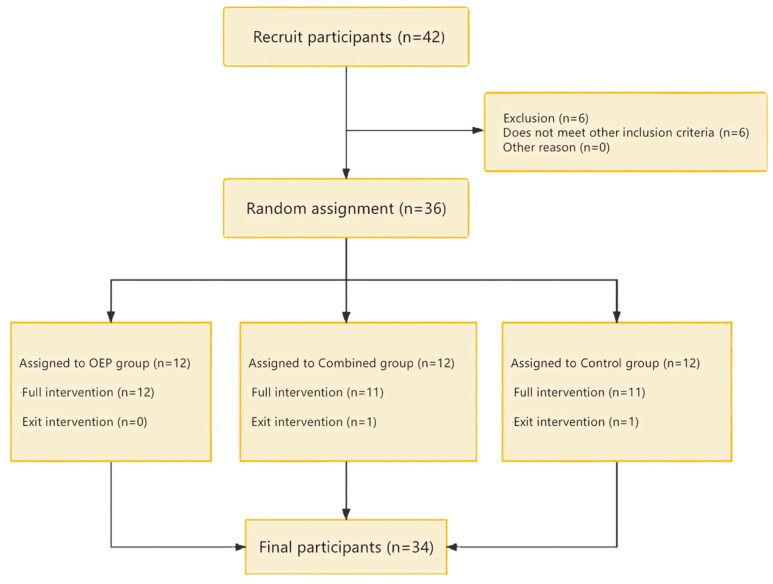
Flow chart of subject recruitment.

Participants for this experiment were recruited from the Taikang Nursing Home in Jinan. After rigorous screening based on the inclusion criteria, 36 participants with ankle instability were initially selected. However, by the third week of the experiment, two participants withdrew for personal reasons (one from the combined group and one from the control group). No adverse events or intervention-related discomfort were reported. All remaining participants completed the prescribed training sessions as scheduled. Therefore, the final data analysis was based on the results of 34 participants (see Figure 5).

Table 2 presents a detailed comparison of the participant information.

In this experiment, participant recruitment, grouping, intervention implementation, data recording, and statistical analysis were carried out by the first and second authors. Basic participant information included age, gender, height, weight, number of sprained ankles, and duration of involvement in specific activities. One-way ANOVA results showed no statistically significant differences in participant information between groups (*p* > 0.05). Specific data can be found in Table 2.

### 3.2. CAIT Score

Repeated measures ANOVA was conducted on the CAIT scores at different time points, leading to the following conclusions: group changes had a significant effect on the CAIT scores (F = 3.354, *p* = 0.048). Time changes also had a significant effect on the CAIT scores (F = 153.481, *p* = 0.000). The interaction between group and time changes also had a significant effect (F = 10.965, *p* = 0.000). The changes in CAIT scores at different time points, as well as the interaction between the group and time points, had significant effects on participants’ CAIT scores.

Within-group comparisons showed that CAIT scores in all three intervention groups significantly improved at both the fourth and eighth weeks compared to pre-intervention (*p* < 0.05). The CAIT scores at the eighth week post-intervention also significantly increased compared to those at the fourth week (*p* < 0.05).

Between-group comparisons revealed significant differences at the eighth week post-intervention between the combined group and the OEP group, as well as between the combined group and the control group (*p* < 0.05). For other groups and time points, no significant changes were observed among the three groups. After 8 weeks of intervention, the average CAIT score in the combined group was higher than in the other two groups. In summary, all three intervention groups effectively improved the instability of the participants’ ankles, with the combined group showing the most prominent effect (Table 3 and Table 4).

### 3.3. VAS Score

Repeated measures ANOVA was conducted on the VAS scores at different time points, leading to the following conclusions: group changes had no significant effect on the VAS scores (F = 0.026, *p* = 0.975). Time changes had a significant effect on the VAS scores (F = 74.975, *p* = 0.000). The interaction between the group and time changes had no significant effect (F = 0.078, *p* = 0.960). The interaction between the group and measurement time points had no significant impact on changes in the participants’ VAS scores.

Within-group comparisons showed that VAS scores in all three groups significantly improved at both the fourth and eighth weeks compared to pre-intervention (*p* < 0.05). However, there were no significant changes in the VAS scores at the eighth week post-intervention compared to the fourth week (*p* > 0.05).

Between-group comparisons showed no significant differences in changes between the combined group and the OEP group, the combined group and the control group, or the OEP group and the control group. In conclusion, while all three groups effectively improved the ankle pain responses of participants, there were no significant differences in the effectiveness between the groups (Table 5 and Table 6).

### 3.4. UST Score

Repeated measures ANOVA was conducted on the UST scores at different time points, leading to the following conclusions: group changes had a significant effect on the UST scores (F = 6.813, *p* = 0.004). Time changes also had a significant effect on the UST scores (F = 217.376, *p* = 0.000). The interaction between the group and time changes also had a significant effect (F = 4.846, *p* = 0.014). Both the measurement time and the group × time interaction significantly influenced UST outcomes.

Within-group comparisons showed that UST scores in all three intervention groups significantly improved at the eighth week compared to both pre-intervention and the fourth week (*p* < 0.05). However, there were no significant changes in the UST scores at the fourth week post-intervention compared to pre-intervention (*p* > 0.05).

Between-group comparisons revealed significant differences at the eighth week post-intervention between the combined group and the OEP group, as well as between the combined group and the control group (*p* < 0.05). No significant differences were detected among the groups at other time points. After 8 weeks, the combined intervention group showed higher UST scores than the other groups. Overall, all interventions improved ankle static balance, with the combined approach demonstrating the greatest improvement (Table 7 and Table 8).

### 3.5. SEBT Score

Repeated measures ANOVA was conducted on the mSEBT scores at different time points, leading to the following conclusions: group changes had no significant effect on the Posteromedial Direction mSEBT scores (F = 0.696, *p* = 0.506). Time changes had a significant effect on the Posteromedial Direction mSEBT scores (F = 78.235, *p* = 0.000). The interaction between group and time changes had a significant effect (F = 22.934, *p* = 0.000). Group changes had no significant effect on the Posterolateral Direction mSEBT scores (F = 1.537, *p* = 0.231). Time changes had a significant effect on the Posterolateral Direction mSEBT scores (F = 108.292, *p* = 0.000). The interaction between group and time changes also had a significant effect (F = 26.118, *p* = 0.000). Group changes had no significant effect on the Anterior Direction mSEBT scores (F = 0.115, *p* = 0.892). Time changes had a significant effect on the Anterior Direction mSEBT scores (F = 62.292, *p* = 0.000). The interaction between the group and time changes had no significant effect on the Anterior Direction mSEBT scores (F = 0.113, *p* = 0.932).

Within-group comparisons showed that for the Posteromedial Direction, both the OEP group and the combined group significantly improved at the eighth week compared to both pre-intervention and the fourth week (*p* < 0.05). The control group significantly improved at the fourth week compared to pre-intervention (*p* < 0.05). For the Posterolateral Direction, both the OEP group and the combined group significantly improved at the eighth week compared to both pre-intervention and the fourth week (*p* < 0.05). For the Anterior Direction, all three groups showed significant improvements at the eighth week compared to both pre-intervention and the fourth week (*p* < 0.05).

Between-group comparisons showed that for the Posteromedial Direction, significant differences were observed between the combined group and the control group, as well as between the OEP group and the control group, at the eighth week post-intervention (*p* < 0.05). For other groups and time points, no significant changes were observed among the three groups. After 8 weeks of intervention, the average mSEBT score in the combined group was higher than in the other two groups.

For the Posterolateral Direction, significant differences were observed between the combined group and the control group, as well as between the OEP group and the control group, at the eighth week post-intervention (*p* < 0.05). For other groups and time points, no significant changes were observed among the three groups. After 8 weeks of intervention, the average mSEBT score in the combined group was higher than in the other two groups.

For the Anterior Direction, no significant differences were observed between the combined group and the OEP group, the combined group and the control group, or the OEP and control groups (*p* > 0.05).

Overall, all interventions improved ankle dynamic balance, with greater effects observed in the combined and OEP groups (Table 9 and Table 10).

## 4. Discussion

This study explored the effects of combining the Otago Exercise Program (OEP) with Neuromuscular Electrical Stimulation (NMES) on chronic ankle instability (CAI) in individuals aged 60 and above. The experimental results indicated that all intervention groups (OEP group, combined group, and control group) showed significant improvements in ankle stability, static balance, and dynamic balance levels post-intervention. Among these, the combined group tended to show greater improvements in several outcomes, especially in the CAIT score, Eyes-closed Single-Leg Stance Test (UST), and Star Excursion Balance Test (mSEBT).

The CAIT scores in all intervention groups significantly improved post-intervention, particularly in the combined group, which showed significant results. This suggests that the combination of Otago Exercise and Neuromuscular Electrical Stimulation was associated with improvements in ankle stability. The CAIT scores at week 8 in the combined group were notably higher than in the other two groups, further validating the significant positive effect of combined intervention on ankle stability and functional recovery. Although pain scores (VAS) in all groups significantly improved at week 4 and week 8 compared to pre-intervention, no significant differences were found between the groups. This suggests that while both Neuromuscular Electrical Stimulation and Otago Exercise have some effect on pain reduction, their impact on pain relief is relatively similar. This may be due to the fact that all interventions involved a certain degree of physical activity and recovery [26]. The static balance (UST) results showed significant improvements in all groups by week 8. Notably, the combined group demonstrated a significant improvement in UST scores post-intervention, surpassing the OEP and control groups. This indicates that the combined intervention may offer additional benefit in enhancing static balance control of the ankle. Dynamic balance testing revealed that the combined group outperformed the OEP and control groups in the posterior-medial and posterior-lateral directions, demonstrating that the combined intervention significantly improves dynamic balance of the ankle joint. The Star Excursion Balance Test (mSEBT), which has been proven as an effective tool for assessing ankle stability [27], further validated its efficacy in evaluating the balance abilities of CAI patients in this study. Dynamic balance assessments such as the modified Star Excursion Balance Test (mSEBT) have demonstrated acceptable reliability and feasibility in older adult populations when appropriately standardized. Previous studies indicate that the simplified mSEBT can be safely performed by older adults and is sensitive to balance-related deficits relevant to functional mobility and fall risk [28]. Therefore, its use in the present study provides a practical and meaningful assessment of dynamic postural control in older adults with chronic ankle instability.

The Otago Exercise Program (OEP) emphasizes improving the neuromuscular control of the ankle joint through a series of balance, gait, and strength training exercises. Through training, older adults can enhance the coordination of the muscles around the ankle joint, particularly the small muscle groups that control ankle movements, thereby improving ankle stability. The training promotes precise control of the muscles by the nervous system through repetitive movement patterns such as single-leg stance and eyes-closed balance exercises, allowing the ankle joint to better adapt to changes in external forces and thereby improving its stability. For example, the calf raises in the OEP effectively strengthen the muscles surrounding the ankle joint, especially the gastrocnemius and soleus muscles [29]. These muscles play a crucial role in maintaining ankle stability. As training continues, the increased muscle strength helps in better support for the ankle joint and reduces the instability caused by muscle weakness [30]. Additionally, strength training in the OEP enhances muscle endurance, allowing patients to maintain better stability during prolonged activity [31]. Furthermore, gait training in the OEP strengthens the neuromuscular feedback loops, enabling faster responses in actual movements [32]. For instance, when there is a slight displacement in the ankle joint, the nervous system can quickly activate the related muscle groups to make adjustments, thus maintaining stability.

NMES enhances muscle endurance and recovery speed by promoting blood circulation and metabolic activity in the muscles [33]. During treatment, NMES improves muscle endurance, allowing the muscles around the ankle joint to maintain effective contractions for a longer period, thereby maintaining stability during prolonged activity. Additionally, electrical stimulation accelerates muscle recovery after fatigue, reducing post-exercise stiffness and discomfort [34]. The deep muscles of the ankle joint are usually less activated during ordinary exercise, but NMES can penetrate deeper muscle layers through electrical stimulation, thus activating these muscle groups [35]. Strengthening deep muscles improves ankle stability, especially in complex movement environments such as walking on uneven surfaces or performing sudden stops and turns [36]. NMES helps restore a more natural and stable gait by promoting normal movement patterns.

In addition to statistical significance, effect size estimates provide important information regarding the magnitude and practical relevance of the observed intervention effects. In this study, the primary outcome (CAIT) demonstrated a large group × time effect (partial η^2^ = 0.414), indicating a substantial and clinically meaningful improvement in perceived ankle stability associated with the combined intervention. Static balance performance (UST) also showed moderate-to-large effect sizes, suggesting meaningful gains in postural control over time. In contrast, effect sizes for pain (VAS) were small, which is consistent with the lack of significant between-group differences and implies that pain reduction may be largely attributable to general activity or time effects. For dynamic balance (mSEBT), effect sizes varied by direction, with larger effects observed in posterior-medial and posterior-lateral reaches, reflecting task-specific improvements rather than uniform changes. Overall, the pattern of effect sizes supports a selective benefit of the combined intervention on ankle stability and balance-related outcomes.

This study shows that the combined intervention (OEP + NMES) significantly outperforms both the OEP-only group and the control group in improving ankle stability, static balance, and dynamic balance. This finding aligns with the effects of single interventions (such as NMES or OEP alone) reported in the existing literature, where most studies have focused on single intervention methods, such as exercise therapy [37], neuromuscular electrical stimulation [38], or traditional rehabilitation treatments [39]. While single interventions can lead to certain therapeutic benefits, due to the physiological and biochemical characteristics of the older adults, single therapies often struggle to achieve optimal results [40]. This study suggests that combining these two methods significantly enhances the effects. This discovery extends current treatment strategies, and for the older adults, combined interventions can help recovery and reduce the risk of injury.

The study also provides a more detailed and comprehensive assessment of ankle stability, involving various aspects such as static balance, dynamic balance, and pain. This offers broader data support for understanding the mechanisms of the effects of different interventions on ankle stability in older adults. Existing studies often measure treatment outcomes using single evaluation criteria, such as pain scores [41] or muscle strength assessments [42], neglecting comprehensive evaluations of ankle joint function, balance ability, and activity levels. This study, however, not only measured ankle stability across multiple dimensions but also combined functional assessments, dynamic balance, and pain response indicators, providing a more thorough understanding of the treatment effects of the combined intervention across different dimensions. This multi-dimensional assessment design makes the study’s results more comprehensive and increases the reliability of the findings.

By combining the Otago Exercise Program and neuromuscular electrical stimulation, this study may represent a feasible and potentially useful rehabilitation approach for older adults with CAI, helping them recover ankle function in a shorter time and reduces the risk of injury. Especially in older patients, whose rehabilitation training is often limited by age and physical ability, combined interventions can achieve better results through low-load exercise.

Importantly, the observed improvements were time-dependent and emerged over the 8-week intervention period, underscoring the significance of the intervention duration when designing and implementing rehabilitation programs for older adults with chronic ankle instability.

## 5. Limitations

However, there are certain limitations in the current study. First, as a pilot study, the sample size was relatively small for a three-group randomized controlled trial, and an a priori sample size calculation/power calculation was not conducted, the sample size was not determined based on a formal power calculation but was intended to assess feasibility and provide preliminary data. This was primarily due to recruitment feasibility constraints in an older nursing home population. Although statistically significant group × time interactions with moderate-to-large effect sizes were observed for key outcomes, the limited sample size may reduce the generalizability of the findings and increase the risk of type II error for some outcomes. Therefore, the results should be interpreted with caution. In addition, participants were recruited from a single nursing home, which may limit the external validity of the findings. Institutionalized older adults may differ from community-dwelling or more physically active populations in functional status, activity levels, and underlying causes of chronic ankle instability. Therefore, the results may not be fully generalizable to older adults with different living environments or etiologies of ankle instability. Furthermore, although validated measures of ankle stability and balance were used, broader functional outcomes such as gait performance, fall incidence or fall risk, and activities of daily living or quality-of-life measures were not assessed. As a result, the findings primarily reflect changes in the impairment and functional performance. Future studies should incorporate more clinically meaningful outcomes to better evaluate the translational impact of the intervention. Fourth, due to the nature of the exercise and electrical stimulation interventions, participant blinding was not feasible, which may have introduced expectation or placebo effects. Although outcome assessors were blinded to group allocation to reduce detection bias, the potential influence of participant expectations cannot be completely excluded. Future trials may consider incorporating attention-control or sham-stimulation conditions to further minimize this source of bias.

Future studies with increased sample sizes should further validate these results and explore the use of this combined approach in diverse populations and ankle instability etiologies.

## 6. Implications for Clinical Practice

The present findings suggest that a combined Otago Exercise Program (OEP) and neuromuscular electrical stimulation (NMES) intervention may be a feasible and safe rehabilitation option for older adults with chronic ankle instability, particularly in institutional or community-based settings. Clinicians may consider this approach to address deficits in ankle stability and balance, as improvements were mainly observed in functional stability and postural control following the 8-week intervention. Pain reduction was comparable across groups, indicating that the added value of NMES may be related more to neuromuscular and balance-related adaptations than to analgesic effects.

From a practical perspective, the intervention is low-cost, non-invasive, and well tolerated by older adults, supporting its potential integration into routine rehabilitation and fall-prevention programs. The time-dependent nature of the observed improvements highlights the importance of a sufficient intervention duration. Future studies with larger samples and longer follow-up are needed to confirm long-term effectiveness and generalizability.

## 7. Conclusions

This randomized controlled trial provides preliminary evidence that an 8-week combined Otago Exercise Program and Neuromuscular Electrical Stimulation intervention may improve ankle stability and balance-related outcomes in older adults with chronic ankle instability and underscores the significance of the intervention duration when designing and implementing rehabilitation programs for older adults with chronic ankle instability. However, these findings are limited to a short intervention period and a single nursing home setting and therefore should be interpreted with caution. Larger, adequately powered trials with longer follow-up periods and more diverse populations are required to confirm these results and to determine the long-term clinical effectiveness and generalizability of the combined intervention before widespread implementation.

## Figures and Tables

**Figure 2:**
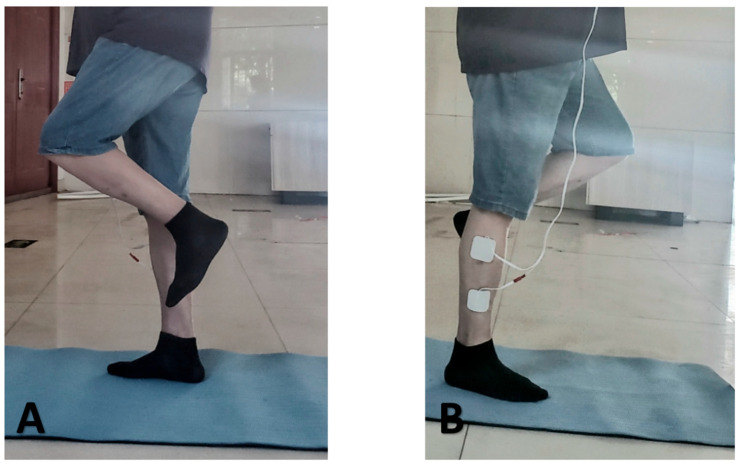
Maintain single-leg stance on a mat. (**A**) the non-NMES side; (**B**) the NMES-applied side.

**Table 2:** Baseline demographic and measurement results of three patient groups.

Variable	OEP Group (*n* = 12)	Combined Group (*n* = 11)	Control Group (*n* = 11)	F	*p*
Age (n)	63.83 ± 3.18	64.00 ± 2.56	63.73 ± 2.93	0.024	0.976
Gender (male/female)	9/3	8/3	7/4		
Height (cm)	171.08 ± 7.99	170.36 ± 7.78	169.27 ± 9.02	0.138	0.871
Weight (kg)	65.67 ± 10.34	64.55 ± 9.93	63.18 ± 10.57	0.168	0.846
Number of Ankle Sprains	0.67 ± 0.65	0.64 ± 0.67	0.73 ± 0.64	0.055	0.947
Left	3	6	7		
Right	9	5	4		

**Table 3:** CAIT score table multiple factors repeated measures ANOVA results.

CAIT Repeated Evaluation of the F-Test			
	F	*p*	Bias η^2^
Group main effect	3.354	0.048	0.178
Time points main effect	153.481	0.000	0.832
Time point × Group	10.965	0.000	0.414

η^2^: partial eta squared (effect size).

**Table 4:** Results of comparison of changes in CAIT score table values with the mean values.

Grouping	Before	4-Week	8-Week	Multiple Comparisons Were Made
	M ± SD	M ± SD	M ± SD	
OEP	5.00 ± 0.76	8.83 ± 0.93 *	12.16 ± 1.15 *	Before < 4-week < 8-Week
Combined	5.64 ± 0.79	9.09 ± 0.97 *	19.00 ± 1.20 * #	Before < 4-week < 8-Week
Control	5.63 ± 0.97	7.54 ± 0.97 *	12.18 ± 1.20 *	Before < 4-week < 8-Week

Note: * there was a significant difference in CAIT scores between before and after intervention (*p* < 0.05) and represents a significant change after the fourth week and 8 weeks after the intervention (*p* < 0.05). # represents a significant change between different intervention groups (*p* < 0.05). M ± SD, represents the mean ± standard deviation.

**Table 5:** VAS table of multiple factors repeated measures ANOVA results.

VAS Repeated Evaluation of the F-Test			
	F	*p*	Bias η^2^
Group main effect	0.026	0.975	0.002
Time points main effect	74.975	0.000	0.707
Time point × Group	0.078	0.960	0.005

η^2^: partial eta squared (effect size).

**Table 6:** Results of comparison of changes in VAS table values with the mean values.

Grouping	Before	4-Week	8-Week	Multiple Comparisons Were Made
	M ± SD	M ± SD	M ± SD	
OEP	4.58 ± 0.67	2.00 ± 0.39 *	1.91 ± 0.35	Before < 4-week < 8-Week
Combined	4.54 ± 0.70	2.18 ± 0.41 *	2.09 ± 0.37	Before < 4-week < 8-Week
Control	4.63 ± 0.70	2.09 ± 0.41 *	2.18 ± 0.37	Before < 4-week < 8-Week

Note: * there was a significant difference in VAS scores between before and after intervention (*p* < 0.05) and represents a significant change after the fourth week and 8 weeks after the intervention (*p* < 0.05).

**Table 7:** UST table of multiple factors repeated measures ANOVA results.

UST Repeated Evaluation of the F-Test			
	F	*p*	Bias η^2^
Group main effect	6.813	0.004	0.305
Time points main effect	217.376	0.000	0.875
Time point × Group	4.846	0.014	0.238

η^2^: partial eta squared (effect size).

**Table 8:** Results of comparison of changes in UST table values with the mean values.

Grouping	Before	4-Week	8-Week	Multiple Comparisons Were Made
	M ± SD	M ± SD	M ± SD	
OEP	23.08 ± 1.89	23.25 ± 1.78	45.67 ± 2.15 *	Before < 4-week < 8-Week
Combined	23.18 ± 1.98	23.91 ± 1.86	60.18 ± 2.25 *#	Before < 4-week < 8-Week
Control	21.18 ± 1.97	20.64 ± 1.86	47.09 ± 2.24 *	Before < 4-week < 8-Week

Note: * UST scores increased significantly from baseline, with significant improvements observed at both week 4 and week 8 (*p* < 0.05). Significant between-group differences were also identified (*p* < 0.05). Values are presented as the mean ± standard deviation (M ± SD). # represents the significant change between different intervention groups (*p* < 0.05). M ± SD, represents the mean ± standard deviation.

**Table 9:** mSEBT table of multiple factors repeated measures ANOVA results.

mSEBT Repeated Evaluation of the F-Test			
Posteromedial Direction	F	*p*	Bias η^2^
Group main effect	0.696	0.506	0.043
Time points main effect	78.235	0.000	0.716
Time point × Group	22.934	0.000	0.597
Posterolateral Direction			
Group main effect	1.537	0.231	0.090
Time points main effect	108.292	0.000	0.777
Time point × Group	26.118	0.000	0.628
Anterior Direction			
Group main effect	0.115	0.892	0.007
Time points main effect	62.095	0.000	0.667
Time point × Group	0.113	0.932	0.007

η^2^: partial eta squared (effect size).

**Table 10:** Results of comparison of changes in mSEBT table values with the mean values.

Grouping	Before	4-Week	8-Week	Multiple Comparisons Were Made
	M ± SD	M ± SD	M ± SD	
OEP(Posteromedial)	71.83 ± 2.43	72.25 ± 2.40	79.41 ± 2.36 * #	Before < 4-week < 8-Week
Combined	70.90 ± 2.54	71.45 ± 2.51	83.63 ± 2.47 * #	Before < 4-week < 8-Week
Control	71.09 ± 2.54	71.90 ± 2.51 *	71.45 ± 2.47	Before < 4-week < 8-Week
OEP(Posterolateral)	67.92 ± 1.86	68.16 ± 1.69	74.25 ± 1.76 * #	Before < 4-week < 8-Week
Combined	68.73 ± 1.94	69.46 ± 1.76	79.27 ± 1.85 * #	Before < 4-week < 8-Week
Control	67.54 ± 1.94	68.18 ± 1.77	68.36 ± 1.85	Before < 4-week < 8-Week
OEP(Anterior)	70.42 ± 1.38	70.33 ± 1.38	78.41 ± 1.14 *	Before < 4-week < 8-Week
Combined	70.54 ± 1.44	71.09 ± 1.44	79.27 ± 1.19 *	Before < 4-week < 8-Week
Control	70.36 ± 1.44	70.64 ± 1.44	77.91 ± 1.20 *	Before < 4-week < 8-Week

Note: * mSEBT scores improved significantly from baseline, with significant gains observed at both week 4 and week 8 (*p* < 0.05). Significant between-group differences were also detected (*p* < 0.05). Data are presented as the mean ± standard deviation (M ± SD). # represents the significant change between different intervention groups (*p* < 0.05). M ± SD, represents the mean ± standard deviation.

## Data Availability

The datasets generated and/or analysed during the current study are available from the corresponding author on reasonable request.
